# Correction to ‘Binding of DNA origami to lipids: maximizing yield and switching via strand displacement’

**DOI:** 10.1093/nar/gkab1163

**Published:** 2021-11-12

**Authors:** Jasleen Kaur Daljit Singh, Esther Darley, Pietro Ridone, James P Gaston, Ali Abbas, Shelley F J Wickham, Matthew A B Baker

**Affiliations:** School of Chemistry, The University of Sydney, Sydney, New South Wales, Australia; School of Chemical and Biomolecular Engineering, The University of Sydney, Sydney, New South Wales, Australia; The University of Sydney Nano Institute, The University of Sydney, Sydney, New South Wales, Australia; School of Biotechnology and Biomolecular Sciences, University of New South Wales, Sydney, Australia; School of Biotechnology and Biomolecular Sciences, University of New South Wales, Sydney, Australia; School of Biotechnology and Biomolecular Sciences, University of New South Wales, Sydney, Australia; School of Chemical and Biomolecular Engineering, The University of Sydney, Sydney, New South Wales, Australia; The University of Sydney Nano Institute, The University of Sydney, Sydney, New South Wales, Australia; School of Chemistry, The University of Sydney, Sydney, New South Wales, Australia; The University of Sydney Nano Institute, The University of Sydney, Sydney, New South Wales, Australia; School of Physics, The University of Sydney, Sydney, New South Wales, Australia; School of Biotechnology and Biomolecular Sciences, University of New South Wales, Sydney, Australia; CSIRO Synthetic Biology Future Science Platform, Brisbane, Australia

The authors wish to correct a source of funding in their article (1).

## References

[1] Singh J.K.D. , Darley E., Ridone P., Gaston J.P., Abbas A., Wickham S.F.J., Baker M.A.B. Binding of DNA origami to lipids: maximizing yield and switching via strand displacement. Nucleic Acids Res. 2021; 49:10835–10850. 34614184 10.1093/nar/gkab888 PMC8565350

